# A New (Old), Invasive Ant in the Hardwood Forests of Eastern North America and Its Potentially Widespread Impacts

**DOI:** 10.1371/journal.pone.0011614

**Published:** 2010-07-21

**Authors:** Benoit Guénard, Robert R. Dunn

**Affiliations:** 1 Department of Biology, North Carolina State University, Raleigh, North Carolina, United States of America; 2 W. M. Keck Center for Behavioral Biology, North Carolina State University, Raleigh, North Carolina, United States of America; Institut Mediterrani d'Estudis Avançats (CSIC/UIB), Spain

## Abstract

Biological invasions represent a serious threat for the conservation of biodiversity in many ecosystems. While many social insect species and in particular ant species have been introduced outside their native ranges, few species have been successful at invading temperate forests. In this study, we document for the first time the relationship between the abundance of the introduced ant, *Pachycondyla chinensis*, in mature forests of North Carolina and the composition, abundance and diversity of native ant species using both a matched pair approach and generalized linear models. Where present, *P. chinensis* was more abundant than all native species combined. The diversity and abundance of native ants in general and many individual species were negatively associated with the presence and abundance of *P. chinensis*. These patterns held regardless of our statistical approach and across spatial scales. Interestingly, while the majority of ant species was strongly and negatively correlated with the abundance and presence of *P. chinensis*, a small subset of ant species larger than *P. chinensis* was either as abundant or even more abundant in invaded than in uninvaded sites. The large geographic range of this ant species combined with its apparent impact on native species make it likely to have cascading consequences on eastern forests in years to come, effects mediated by the specifics of its life history which is very different from those of other invasive ants. The apparent ecological impacts of *P. chinensis* are in addition to public health concerns associated with this species due to its sometimes, deadly sting.

## Introduction

Ants are among the most economically [1], [2], [3] and ecologically significant groups of biological invaders [4], [5], [6], [7], [8], [9]. However, despite the introduction of hundreds of ant species outside of their native ranges and considerable research only a handful of introduced ant species have been shown to have clear negative effects on native ant species. Moreover, most studies on invasive ants are conducted in heavily disturbed habitats. In contrast, relatively few studies have examined the influence of invasive ants on native ant diversity in undisturbed or relatively undisturbed habitats and these studies primarily come from island ecosystems [10], [11], tropical ecosystems [10], [12], [13] or temperate ecosystems such as riparian corridors and fire-adapted grasslands and woodlands [14], [15], [16], [17], [18] which have a high frequency of natural disturbance. To the extent that the literature on invasive ants characterizes our understanding of them, it seems that in the temperate and tropical, mainland, forests of the world invasive ants are minor players.

As with other taxa including plants, birds, mammals and fish (reviewed in [19]), highly invasive ants are relatively restricted in their taxonomic and biogeographic distributions of origin [20]. Although there are 22 subfamilies of ants, the most widespread and damaging ant invaders come from the three most diverse and, arguably, ecologically dominant subfamilies ([21]; Dolichoderinae, Formicinae, and Myrmicinae), and originate from sub-tropical or tropical regions [6]. For example, although tens of species from the diverse subfamily Ponerinae have been introduced outside their native range [6], [22], [23], [24], [25], no species from this subfamily have been recognized as invasive.

Recently, a non-native ant species in the ponerine genus *Pachycondyla* has been found to be common in parts of the southeastern U.S. [26]. In contrast to the other diverse ant subfamilies, species of the subfamily Ponerinae, , have often been described as possessing both morphological and behavioral traits thought of as “basal” [21], [27]. Although it is clearly abundant, it is not clear whether *Pachycondyla (Brachyponera) chinensis* (Emery) meets the requirement of being invasive as we define it here of having negative ecological effects on native species. If it does, *P. chinensis* represents an interesting exception to the rules of ant invasion in being from the Ponerinae subfamily, invading hardwood forests and originating from cold-temperate regions. Due to its potent sting, this species has been identified as an emerging public health threat [26]. However, no research has considered its geographic distribution or the ecological consequences of its invasion.

Here we examine the changes in the diversity of native ant assemblages and the abundance of key native ant species associated with the invasion of *P. chinensis* in old forests of Eastern North America. We surveyed ant communities in presence and absence of *P. chinensis* in 50 plots at 25 sites in five different forest landscapes in Wake County, North Carolina. Across these plots, we tested the hypothesis that the presence and abundance of *P. chinensis* is negatively associated with the diversity (measured as species density) of native ant assemblages and the abundances of native species. We measured the effect of *P. chinensis* presence and abundance on native ant communities at pitfall, local, and landscape scales. We examine these relationships overall and then separately for key taxonomic and functional groups (*Camponotus spp.*, seed dispersers, specialist litter foraging ants…), in order to determine whether there are particular functional groups whose absence is likely to lead to cascading ecological effects on ecosystem processes. In addition, we compare the diversity of ants found in sites with and without *P. chinensis* to samples from forests around the world to test whether the diversity of sites with *P. chinensis* is unusual even in a global context. To our knowledge, this is the first study of the consequences of an invasive ant in a mature temperate forest ecosystem. While none of the forests in eastern North America are pristine [28], our study sites are typical of mature temperate forests from North Georgia to southern Massachusetts and so any consequences of *P. chinensis* in these forests have geographically broad implications.

## Methods

### Study Organism

What is known about *P. chinensis* is fragmentary. It appears to have been introduced to the United States no later than the 1930's from Japan [29], [30]. It was described in the beginning of the last century as having small, inconspicuous colonies [29]. Since then it has only been studied once, and even then only in the context of its potential public health threat [26]. Many reports have been noted of humans suffering anaphylactic shock or dermatosis after being stung by *P. chinensis* both in its native [31], [32], [33], [34], [35] and introduced ranges [36]. The lack of study of *P. chinensis* is not because it is rare. Our recent work suggests that the species is now distributed in no fewer (and likely many more) than nine states in the Eastern North American coast, from Connecticut to the northernmost part of Florida (Figure S1). Further, where it is present, *P.chinensis* has been found in anthropogenic habitats, such as city sidewalks and backyards [29], [37] and agricultural habitats [38] but is particularly abundant in mature, temperate, hardwood forests, including a national park (Great Smokey Mountains National Park) and several state parks within North Carolina, South Carolina (unpublished data), and Alabama [39]. Given the distribution of this species, whatever its effects on native ant and other species, they are likely to be widespread.

### Ethics statement

This work was conducted according to relevant national and international guidelines.

### Study sites

This study was conducted in five mature closed-canopy, mesic deciduous forests of Wake County, North Carolina, USA. The five forest will be referred as 1) North Carolina State University fragment forest (NCSU), 2) Yates Mill Pond forest (YMP), 3) Schenck Memorial forest (SM), 4) Hemlock Bluff forest (HB), 5) and Cary remnant forest (CRF). Two of the forests are remnants of larger forest now situated in urban development (NCSU and CRF), while the three others are larger protected forests (YMP, SM and HB).

Each site consisted of two plots of 16 pitfall traps installed on a square grid of 15×15 m side (total area 225 m^2^); with pitfall traps separated by 5 meters. Pitfalls traps had a diameter of 6.2 cm, and were filled with 3 cm of antifreeze liquid as preservative. The two plots within a site were placed so that one would have *P. chinensis* present and the other would not. The “invaded” plot was installed where we directly observed *P. chinensis* foragers or nests. Once the invaded plot was installed, we installed a second plot (within 20–100m of the invaded plot) where we observed no *P. chinensis* nests or foragers. The second treatment (hereafter “non-invaded plots”) had similar vegetation cover, tree species composition, dead wood, and slope exposure as the paired invaded plot. Thus the sampling design was matched pair with an invaded and non-invaded plot. Matched pair designs while not fully experimental, have the advantage of controlling for environmental factors that vary among sites independent of the “treatment” of interest.

We installed 21 sites for a total of 672 pitfall traps. All pitfalls were active for 72 hours in the months of June–July in 2007 and 2008. Sites were located within the five chosen forests in the following pattern, two sites at NCSU (one site in 2007 and 2008); eight sites at YMP (five in 2007 and three in 2008); two sites at SM in 2007; eight sites at HB (seven in 2007 and one in 2008); one site at CRF in 2008. After 72 hours, the pitfall traps were collected. Ants were sorted, identified to morphospecies and counted. Vouchers were deposited in Rob Dunn laboratory's collection and in the NC State University Insect Museum.

### Species richness estimates at the landscape scale for areas with and without *P. chinensis*

Individual data from pitfall traps were separated into two groups based on the presence or absence of *P. chinensis* within each pitfall trap. For each group, we analyzed the data to evaluate the species accumulation over our sampling effort with the software Estimate S [40]. We used Chao1 as estimator for each group of the total species richness in both invaded and non-invaded areas.

### Association between *P. chinensis* and native species abundance at a local and site scale

#### Local scale

We used an ANOVA to compare the effect of the treatment, invaded vs. non-invaded, on the total ant abundance and on native ant abundance at the pitfall trap scale. All abundance data were Log(x+1) transformed to achieve homogeneity of variance both for this and subsequent tests.

For a few plots, a small subset of pitfall traps in the “non-invaded” treatment was actually found to contain *P. chinensis*. In a second ANOVA, we considered at a pitfall trap scale the actual presence or absence of *P. chinensis* and its effect on native ant abundance. Pitfall traps were separated into two categories: those without any *P. chinensis* individual per pitfall trap, and those with at least one individual collected per pitfall trap.

#### Site scale

To compare native ant abundance between treatments among sites, we standardized the sampling effort by considering the the number of ants collected per pitfall trap. Homogenization was necessary due to pitfall removal by macrofauna (3% in 2007 and 18% in 2008). We then performed an ANOVA with site as a block effect. A Generalized Linear Model was realized to predict the response of native ant species abundance. The variables used in the model were native species density, the number of pitfall trap per site, abundance of *P. chinensis* and the saturation of *P. chinensis* per site (number of pitfall traps per site where *P. chinensis* was collected). This method has two advantages over an ANOVA or an ordinary least squares regression. First, it allows us to keep our full set of data, even sites where one but not all pitfall traps in a control site had *P. chinensis*. Second, it allows us to predict the native ant species abundance or species density response to variation in the other variables entered into the model.

### Association between *P. chinensis* and native species density at the local and site scales

#### Local scale

We used an ANOVA to compare the effect of the treatment; invaded versus non-invaded, on the native ant species density (our measure of species diversity) at the pitfall trap scale. Data were first analyzed in the context of the matched pair design and then according to the actual presence of *P. chinensis* within the pitfall trap.

#### Site scale

We first compare species density among treatments according to the matched pair design. To homogenize our sampling effort among treatments and among sites, we randomly removed pitfall traps to obtain a number of 13 pitfall traps per treatment. Sites that had suffered high pitfall trap removal rates by vertebrates and had less than 13 pitfall traps per plots have not been considered in this analysis. A total of sixteen sites have been kept for this analysis (five sites in 2008 had less than 10 pitfall traps left for at least one of their treatment). We used an ANOVA with site number as a block effect. A Generalized Linear Model was realized to predict the native ant species density at the site scale. The variables used in the model were native species abundance, the number of pitfall traps per site, abundance of *P. chinensis* and the saturation of *P. chinensis* per site.

### Association between *P. chinensis* abundance and native species groups

To measure the response of native ant species to the abundance of *P. chinensis* we consider the abundance of *P. chinensis* for each pitfall trap and its related native species density and abundance. To do so, we consider as a control group the pitfall traps where no *P. chinensis* have been collected for an entire plot. The pitfall traps with no *P. chinensis* but for which at least one individual of *P. chinensis* have been collected by one of the pitfall traps installed at a plot were considered as a “0” group. For the rest of the pitfall traps, the categories based on *P. chinensis* abundance have been established (1–5, 6–10, 11–20, 21–50, and 51–500). Total native ants' species density was considered, as the effect on specific taxa/group. Groups of species were based mainly on taxonomical relationship. However in order to include species with natural low densities, we also create groups based on ecological relationship. Species collected for a specific genus, tribe or functional group were considered together. Those were: *Aphaenogaster*, the main seed dispersal ants (7 species), *Camponotus* (7 species), *Crematogaster* (5 species), *Formica* (3 species), the small-medium sized Formicinae: *Brachymyrmex*, *Lasius*, *Nylanderia* and *Prenolepis* (10 species), the small generalist Myrmicinae: *Monomorium*, *Solenopsis* and *Temnothorax* (8 species), and the hypogaeic ants, also referred as leaf litter foragers: *Amblyopone*, *Hypoponera*, *Myrmecina*, *Ponera*, *Pyramica*, *Strumigenys* (10 species). This last group is essentially composed of predator specialist of small arthropods such as Collembolan, Acari, or Chilopods (Traniello 1982, Masuko 1984, 1994, 2009a, b). To compare the effect of the abundance of *P. chinensis* on the different groups of ants, an ANOVA was realized among the different categories, and if this test was significant, multiple comparisons were realized with the Tukey's HSD test, which consider overall error rate in multiple comparisons.

### The association between *P. chinensis* and leaf-litter ants

While pitfall traps are efficient to measure ground foraging ants, they are less effective at capturing litter dwelling (hypogaeic) ant species. In contrast, Winkler litter extractors are useful for sampling those species that nest and forage within leaf litter and so provide a complementary picture of ant assemblages to that derived from pitfall traps [41], [42]. In July 2009, we monitored ant communities with a focus on hypogaeic ants with the use of Winkler bags extractors. We selected four new sites at Yates Mill Pond Forest composed of two plots, each of 400 m^2^, one invaded by *P. chinensis* and one non-invaded. Again, plots were chosen according to a matched-pair design and were separated from each other by 20 to 50 m. Leaf litter was collected at least at 12 locations within each plot to reach a volume of five liters of sifter leaf-litter material [43]. Collection within each plot was done so as to maximize the diversity of micro-sites chosen (leaf litter from base of tree, near large size log, deep humus area…) and capture as many species as possible. After collection, leaf litter was dried with the Winkler extractor technique for five days. Ant species density and abundance per plot were compared with a block ANOVA, with site number as a block effect.

Finally, we compared our data from Winkler sampling with those published in Ward [44] to understand the effect of ant abundance and presence absence of *P. chinensis* on species density. We used a stepwise model to determinate the importance of ant abundance (after log transformation) and *P. chinensis* presence or absence on species density.

## Results

Out of 672 installed pitfall traps, 614 pitfall traps were collected (58 were removed by vertebrates). A total of 14,437 individuals (11,270 in 2007 and 3,167 individuals in 2008) representing 52 species were collected (Table S1). At least one individual of *P. chinensis* was found in each of 306 pitfall traps. *P. chinensis* was absent from 308 pitfalls.

Under the matched-pair design, 36 native species were recorded in invaded areas, while 48 species were recorded in non-invaded areas. Chao1 estimates of richness were only slightly higher (39 and 57.2 species in the invaded and non-invaded areas respectively) than observed numbers of species suggesting that our sampling captured most ant species possible given the methods used. Our sampling completeness is estimated at ≈91% for invaded areas and ≈84% for non-invaded areas of the species sampleable given the methods.

### Association between *P. chinensis* and native species abundance at a local and site scales

#### Local scale (Pitfalls)

Ant abundance (the total number of individual ants of all species per sample) was more than twice as high in invaded plots (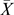
_invaded_ = 32.3±48.3) than in non-invaded plots (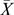
_non-invaded_ = 14.2±14.3) (*F*_[1–612]_ = 83.11; *p*<0.0001) due to the abundance of *P. chinensis*. The abundance of native ants actually showed the opposite pattern, with many more individuals in non-invaded than in invaded plots (*F*_[1–612]_ = 99; *p*<0.0001). The abundance of individuals of native species was almost twice as high in non-invaded plots (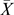
_non-invaded_ = 14.2±14.3) as in invaded plots (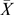
_invaded_ = 7.1±8.1). When presence/absence of *P. chinensis* was considered, a similar pattern was observed (*F*_[1–612]_ = 148.1; *p*<0.0001). The number of individuals of native species was more than two times higher in non-invaded plots (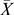
_invaded_ = 6.5±7.7; 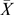
_non-invaded_ = 14.3±14.1).

**Figure 1:**
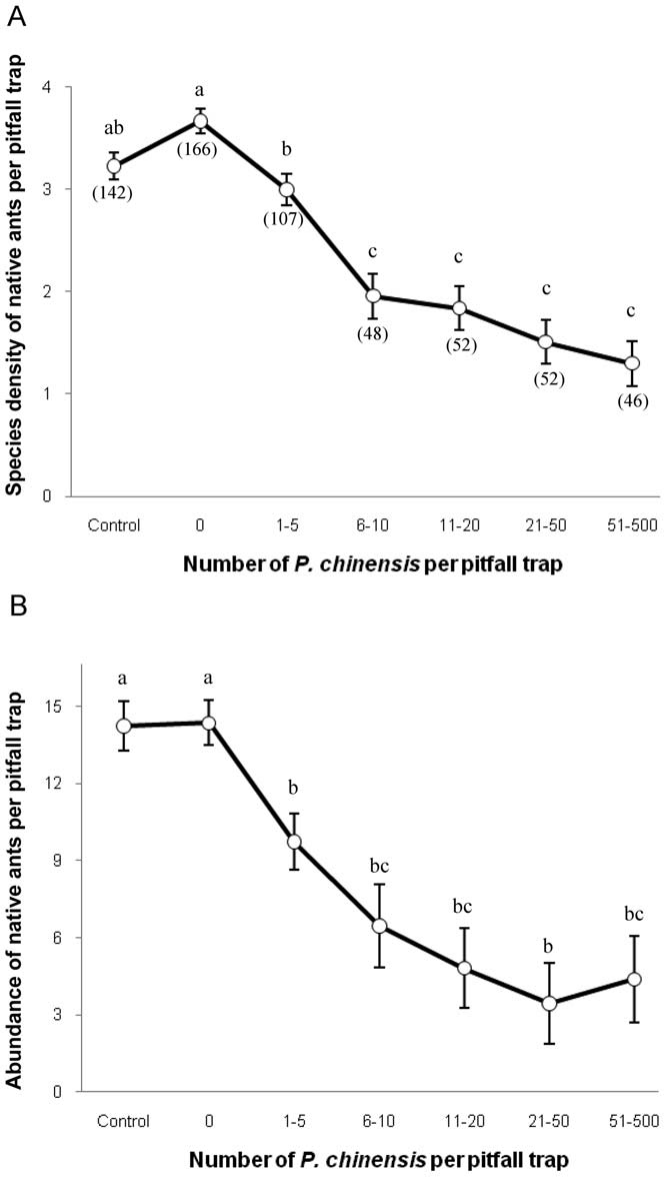
Responses of the species density and abundance of native ants to *P. chinensis* abundance. (A) Relationship between the abundance of *P.chinensis* and the species density of native ants per pitfall trap. (B) Relationship between the abundance of *P.chinensis* and the abundance of native ants per pitfall trap. Numbers in parenthesis represent the number of pitfall traps for each category.

The abundance of native ant was negatively associated (F_[6–606]_ = 14.69; *p*<0.0001) with the abundance of *P. chinensis* at the local scale (Fig 1B). The mean abundance of native species captured in pitfall traps without *P. chinensis* was 2 to 3 times higher than on pitfall traps where *P. chinensis* abundance was high (more than 20 *P. chinensis* per pitfall trap).

#### Site scale

Mean total ant abundance per site was higher in invaded sites (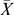
_invaded_ = 32.1±15.2) than for non-invaded plots (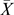
_non-invaded_ = 14.3±15.1) (*F*_[1–20]_ = 11.95; *p* = 0.0025). However, mean native ant abundance per site was two times lower within invaded plots (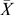
_invaded_ = 7.1±3) than in non-invaded plots (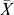
_non-invaded_ = 13.8±3) (*F*_[1–20]_ = 19.56; *p* = 0.0003). Block effect were not significant whether for all ants (*p* = 0. 43) or when just native species were considered (*p* = 0. 13).

The results of the Generalized Linear Model (Table 1) indicated that in addition to the overall abundance of *P. chinensis* in a plot, the coverage of *P. chinensis* within a plot (number of pitfall traps with *P. chinensis* for a given plot) had a negative effect on native species abundance. All else equal, for example, the complete coverage of *P.chinensis* across a site (*P. chinensis*) was associated with a reduction in the abundance of native ants collected per pitfall trap by 10 individuals relative to a site where *P. chinensis* is absent.

**Table 1:** Four nested Generalized Linear Models of native ant abundance.

	Native species density		+ Number of pitfall traps per site		+ *P. chinensis* abundance		+ Saturation of *P. chinensis*	
	Parameter estimates	Effect test	Parameter estimates	Effect test	Parameter estimates	Effect test	Parameter estimates	Effect test
**Intercept**	3.62	***P***<0.0001	3.18	***P***<0.0001	3.30	***P***<0.0001	3.32	***P***<0.0001
**Native species density**	0.12	***P***<0.0001	0.11	***P***<0.0001	9.7×10^−2^	***P***<0.0001	8.1×10^−2^	***P***<0.0001
**Number of pitfall trap per site**	Not included	Not included	3.3×10^−2^	***P***<0.0001	4.3×10^−2^	***P***<0.0001	6.3×10^−2^	***P***<0.0001
***P. chinensis*** ** abundance**	Not included	Not included	Not included	Not included	−4.0×10^−4^	***P***<0.0001	−1.5×10^−4^	***P***<0.0001
**Saturation of ** ***P. chinensis***	Not included	Not included	Not included	Not included	Not included	Not included	−2.8×10^−2^	***P***<0.0001
**−Log likelihood**	571.26		584.4		607.8		664.6	

Models are arranged according to increasing complexity, from left to right. The first model includes only the native species density. “+number of pitfall traps per site” includes the number of native species as well as the number of pitfall traps collected per site. “+*P. chinensis* abundance” model includes the number of native species, the number of pitfall traps per site and the total abundance of *P. chinensis* per site. “+Saturation of *P. chinensis*” model adds the effect of the number of pitfall traps collected where *P. chinensis* was present. Note that the effects of native species density and number of pitfall traps are positive on native species abundance, while both the effects of abundance and saturation of *P. chinensis* are negative on native species abundance. All more complex models are significantly better using −log likelihood ratio than the simpler model. All four models are exponentials of the form *species abundance* = *e* ^Σ parameter i*value *j*^.

### Association between *P. chinensis* and native species density at a local and site scale

#### Local scale (Pitfalls)

Native ant species density was significantly lower within pitfall traps in invaded plots (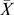
_invaded_ = 2.28±1.63) than in non-invaded plots (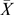
_non-invaded_ = 3.34±1.65) (*F*_[1–612]_ = 77.35; *p*<0.0001). When actual *P. chinensis* presence/absence was considered for the local scale, a similar but more pronounced pattern was observed (*F*_[1–612]_ = 119.9; *p*<0.0001). Native species density was lower in presence of *P. chinensis* (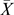
_invaded_ = 2.1±1.53) than in its absence (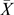
_non-invaded_ = 3.5±1.66).

The density of native ant species (F_[6–606]_ = 30.69; *p*<0.0001) was negatively associated with the abundance of *P. chinensis* at the local scale (Fig 1A). No significant differences in native species density have been observed between pitfall traps without *P. chinensis* but on sites where *P. chinensis* was present (category “0”) and the pitfall traps on control sites.

#### Site scale

Native ant species density was lower in invaded plots (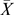
_invaded_ = 8.9±2.1) than in non-invaded plots (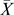
_non-invaded_ = 13.1±2.1) (*F*_[1–15]_ = 16.48; *p* = 0.001). The block effect of the site was not significant (*p* = 0.21).

In the Generalized Linear Model (Table 2) the abundance of *P. chinensis* was negatively associated with native ant species density.

**Table 2:** Four nested Generalized Linear Models of species density of native ants.

	Abundance of native ants		+ Number of pitfall traps per site		+ *P. chinensis* abundance		+ Saturation of *P. chinensis*	
	Parameter estimates	Effect test	Parameter estimates	Effect test	Parameter estimates	Effect test	Parameter estimates	Effect test
**Intercept**	2.06	***P***<0.0001	2.01	***P***<0.0001	1.99	***P***<0.0001	1.96	***P***<0.0001
**Abundance of native ants**	2.1×10^−3^	***P***<0.0001	2.1×10^−3^	***P***<0.0001	1.4×10^−3^	***P*** = 0.0091	1.2×10^−3^	***P*** = 0.045
**Number of pitfall trap per site**	Not included	Not included	3.4×10^−3^	Not significant	1.8×10^−2^	Not significant	2.5×10^−2^	Not significant
***P. chinensis*** ** abundance**	Not included	Not included	Not included	Not included	−5.3×10^−4^	***P*** = 0.0021	−4.5×10^−4^	***P*** = 0.016
**Saturation of ** ***P. chinensis***	Not included	Not included	Not included	Not included	Not included	Not included	−9.2×10^−3^	Not significant
**−Log likelihood**	10.67		12.20		15.43		15.85	

Models are ordered according to increasing complexity, from left to right. The first model includes only the abundance of native ants. “+number of pitfall traps per site” includes the abundance of native ants as well as the number of pitfall traps collected per site. “+*P. chinensis* abundance” model includes the abundance of native ants, the number of pitfall traps per site and the total abundance of *P. chinensis* per site. “+saturation of *P. chinensis*” model includes the effect of the number of pitfall traps collected where *P. chinensis* was present. Only the effect of the abundance of native ants is positively associated with native species density, while only *P. chinensis* abundance is negatively associated with native species density. All four models are exponentials of the form *species density* = *e* ^Σ parameter i*value *j*^.

### Association between *P. chinensis* abundance and native ant species groups

**Figure 2:**
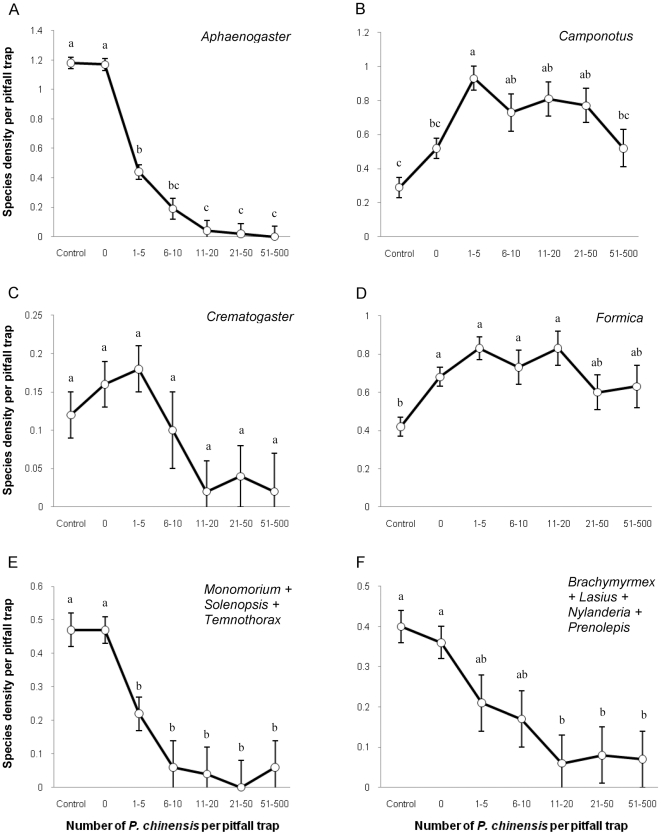
Relationship between *P. chinensis* abundance and the native species density. (A) *Aphaenogaster* (B) *Camponotus* (C) *Crematogaster* (D) *Formica* (E) small Myrmicinae, and (F) small Formicinae.

Species of *Aphaenogaster*, the small Myrmicinae, the small Formicinae and the leaf litter foraging ants, showed no difference between the control and the “0” treatment (*P. chinensis* not collected in the pitfall but present in the plot), but show a strong (negative) association with the abundance of *P. chinensis* (Fig 2 I,V, VI,VII). Species of the genus *Aphaenogaster*, one of the most common groups of species in absence of *P. chinensis* were absent where *P. chinensis* reached high abundances. *Crematogaster* species density, in turn, decreased with increases in *P. chinensis* abundance, though non-significantly (Fig 2 III). Species densities of *Camponotus* and *Formica* increased with increases of the abundance of *P. chinensis*, except where *P. chinensis* was at its most dense (Fig 2 II,IV).

### Association between *P. chinensis* and leaf-litter ants

Leaf litter samples yielded 1923 ants from 27 species. Only 16 individuals of 2 native ant species were collected in invaded plots, while 1347 individuals of 25 species were collected in non-invaded plots.

The abundance of native species was significantly lower in invaded plots (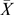
_invaded_ = 4±83.36) than in non-invaded plots (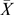
_non-invaded_ = 336.75±83.36) (*F*_[1–3]_ = 39.34; *p* = 0.0082). Native ant species density was significantly lower in invaded plots (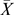
_invaded_ = 0.75±1.41) than in non-invaded plots (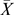
_non-invaded_ = 11.75±1.41) (*F*_[1–3]_ = 53.13; *p* = 0.0053). The block effect of the site was not significant (*p* = 0.61) or species density (*p* = 0.44).

**Figure 3:**
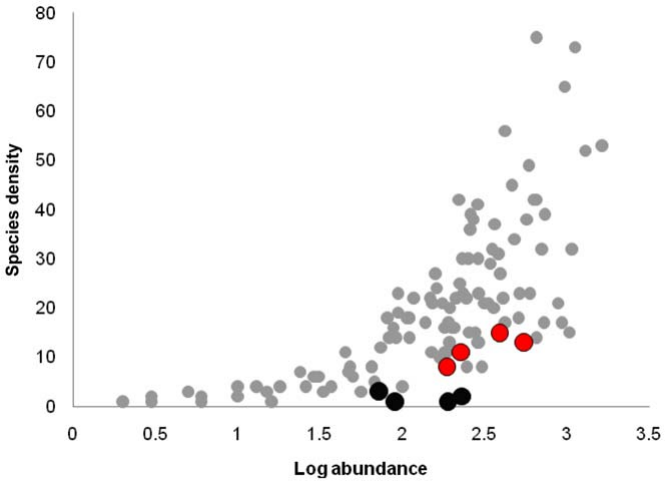
Ant species density as a function of the number of individuals collected with Winkler techniques. Grey circles represent sites presented in Ward (2000) and our own sampling using Ward's method (site details in supplement). Red circles represent sites sampled within North Carolina where *P. chinensis* was absent. Black circles represent sites collected in North Carolina where *P. chinensis* was present.

When the sites we sampled for litter ants were considered in a global context, ant species density was positively correlated with both log-abundances of ant collected (R^2^ = 0.46; *p*<0.00001) and negatively correlated with *P. chinensis* presence (R^2^ = 0.08; *p*<0.00001). Sites with *P. chinenis* in North America had low species abundance given the number of individuals they included and more generally compared to other sites, even those at much higher latitudes or elevations (Fig 3).

## Discussion

**Figure 4:**
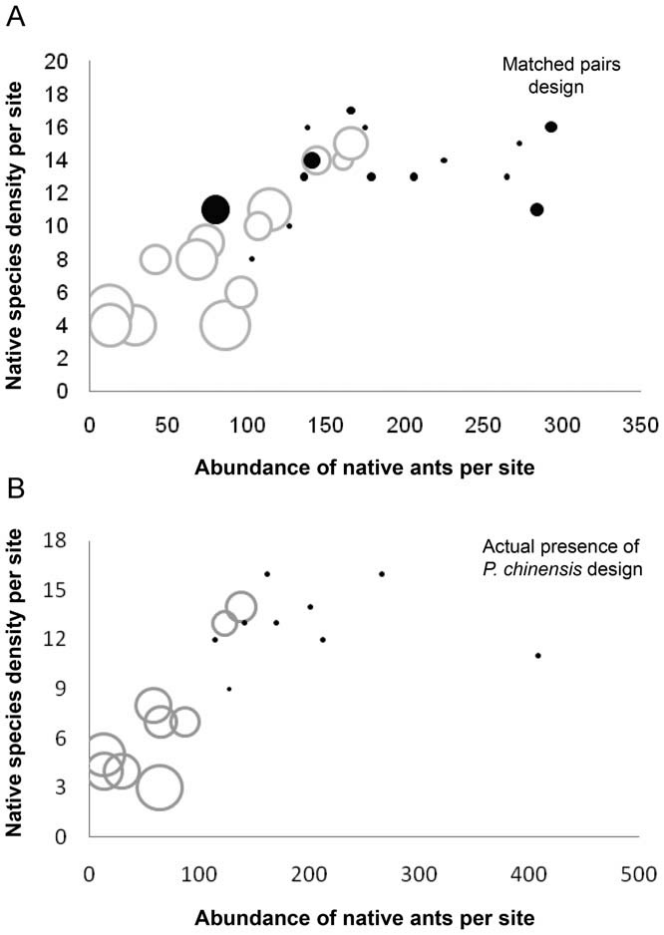
Species density of native ants per site as a function of the abundance of native ants and the presence of *P. chinensis*. Sites with *P. chinensis* are represented by grey circles and sites without *P. chinensis* are in black. Width of circles is relative to the abundance of *P. chinensis* found per site (after data transformed with a log +2). (A) Matched pair design (15 paired sites of 13 pitfall traps used for each site). (B) Actual presence of *P. chinensis* design (9 paired sites with 12 pitfall traps used for each site).

While invasive ants are often associated with a strong disruption of the abundance and diversity of native ants [10], [15], [16], [17], [45], [46], [47], [48], [49], [50], [51], [52], such effects have seldom been documented in mature, hardwood forests, be they temperate or tropical. In our study of mature forests, the presence of *P. chinensis* was negatively correlated with both abundance and native ant species density at each of the scales considered. Furthermore the abundance of several native ant species was strongly negatively associated with increasing densities of *P. chinensis* (Fig 4). We suggest that *P. chinensis* be regarded as an invasive species on the basis of its abundance alone, but also its apparent impacts, expansion in range over the last 80 years [39], and known public health threat [26], [36].

*Pachycondyla chinensis* presence and increasing abundance were associated with lower native ant abundance at both local (pitfall traps grain) and site scales. For all scales considered, the native species abundance in areas with *P. chinensis* was half as great as that in control plots for all designs and scales considered. Where present, *P. chinensis* accounts for 75% of the overall abundance of ants collected; and its abundance is two times higher than the abundance of all native ants collected in non-invaded areas. The ability of invasive species to reach larger abundance than native species for similar habitat has been reported for the big-headed ant *Pheidole megacephala* [13], the Argentine ant, *Linepithema humile* [14], [53], and the fire ant *Solenopsis invicta* [46]. While the exact mechanisms that allow invasive ants to sustain such abundance remain unknown, several authors have noticed the ability of invasive ants to tend Homopteran insects or to exploit sweet-secretions from plants to obtain carbohydrate resources [13], [16], [54], [55]. Hypothesis linking invasion success by ants and a sugar-rich diet have been proposed [6], [52], [54], [56], [57], [58], [59]. In the case of the invasion by *P. chinensis*, this mechanism seems unlikely. Foragers are very rarely observed on vegetation, and are essentially unable to climb trees or vertical surfaces (B. Guénard, *personal observation*), likely as a function of an absent or reduced arolium structure as is the case for most ground-dwelling *Pachycondyla* species [60].

However, even though *P. chinensis* does not appear to benefit from sugar sources, the availability of key dietary resources may be important. *P. chinensis* has been described as a termite specialist in its native range [61], [62] and observations to date suggest that termite and other insects, including native ant species, are also important in its introduced range [26] (B. Guénard, *personal observation*). Furthermore, nests of *P. chinensis* are often found in vicinity or within colonies of termites [62] (B. Guénard, *personal observation*). Colony densities of the termite *Reticulitermes flavipes*, for example, can be extremely high, reaching 300 colonies per hectare within forests close to our sites [63]. Colonies of *R. flavipes* contain between 25 000 [64] to 365 000 individuals [65] such that conservative estimates suggest several million termites may be present in an acre of temperate forests typical of our study sites. If *P. chinensis* is better able to harvest termite resources than native ants, termites may serve to increase the total energy diverted to ants in temperate forests. Future studies could usefully focus on the nesting and foraging abilities of *P. chinensis* relative to termite nests and their inhabitants, the effects of termite resources on *P. chinensis* colony growth and behavior, and the direct and indirect effects of *P. chinensis* on termite densities and decomposition rates within forests.

Like abundance, the species density of native ants was negatively correlated with increases in the abundance of *P. chinensis*. Native ant species collected in invaded areas with pitfall traps were in average 30 to 40% less diverse than in non-invaded areas. At the landscape scale, eighteen native species, 30.5% of the total, have never been collected in invaded areas (Tables S1 and S2). As a consequence, Chao 1 estimates of total species richness are 32% lower in invaded areas. In addition, twelve species (23%) present in invaded areas had their abundance reduced by at least an order of magnitude, if not two. Collectively, more than half of the species we collected were negatively associated with the presence of *P. chinensis*. Furthermore, native ant species density was strongly negatively correlated with increases in the abundance of *P. chinensis* (Fig 2), suggesting a direct effect between *P. chinensis* densities and the measured effects on native ants. The Argentine ants are associated with similar changes in native ant communities in upland coastal habitats in California [17].

Interestingly, many but not all native ant species were rarer where *P. chinensis* was more common. A strong negative relationship between the abundance of *P. chinensis* and species of the keystone seed dispersing ant (*Aphaenogaster* species) was observed, with similar patterns for the small species of the subfamilies Formicinae and Myrmicinae, and litter foraging ant species. For those groups, the species density collected for the control areas and the “0” areas was similar, but was lower with the presence and increasing density of *P. chinensis*. In contrast, species density for larger species from the genera *Camponotus* and *Formica* responded positively for low to medium abundance of *P. chinensis*, and had similar species density to the control areas even for large densities of *P. chinensis*. Tolerance to invasive species by a subset of native species is known for the fire ant, *S. invicta* [66], [67], [68], [69], *Anoplolepis gracilipes* [11], and the Argentine ant, *L. humile* [14], [17], [43], [49], [53]. Mechanisms suggested to explain which species persist alongside invasives are typically based on temporal-seasonal [17], [43], [53] or spatial avoidance of the invasive species [17]. However, the traits that allow species to persist in the presence of an invasive are likely to depend on the life history of the invasive. In sites where fire ants and Argentine ants are present, hypogaeic species persist by foraging in microsites where the invasives are unlikely to encounter them [14], [43], [52], [53]. In contrast, in our study hypogaeic species were strongly negatively correlated with *P. chinensis* presence (Table S2), perhaps for the simple reason that *P. chinensis* is itself more of a hypogaeic forager than either Argentine ants or fire ants. In contrast, the species that persist with *P. chinensis* are larger-bodied *Camponotus* and *Formica*. Why these species survived, where others did not is unclear.

A key question in light of the strong correlations between the *P. chinensis* abundance and presence and the composition of native ant communities is whether *P. chinensis* tends to invade sites with few native ants or whether it leads to the decline in native ant abundance. While several recent studies on fire ant tend to suggest that some invasive ants tend to invade where diversity is already low [70], [71], [72], at least for the regions considered, studies following the progression of invasive species and the associated reduction of native species through time in natural habitat of Australia on *Pheidole megacephala* [13] or on Argentine ant in California [52] lend to support to the idea that invasive ants can have direct impacts on native ants over short time scales. Our study was not experimental and so we cannot say with absolute certainly which of these two mechanisms is at play with *P. chinensis*. However, several lines of evidence suggest that *P. chinensis* is actually driving native ant diversity rather than the other way around. First, most of our sites were located in protected forests where recent human disturbances have been marginal, such that low ant diversity in invaded sites due to disturbance is unlikely. Second, our matched pair design accounts, to the extent possible, for environmental differences between invaded and uninvaded sites by comparing similar, adjacent, sites. In this regard, if the presence of specific species in our study provides information on the quality of habitat, the absence of exotic or native open-habitat specialists in our sampling is also relevant. Invasive and exotic species like fire ant, the Argentine ant, or the pavement ant (*Tetramorium caespitum*) are common species in open-disturbed habitat of Wake county, North Carolina [59], [73], [74], and are well established in open-areas around the forests sites we used (B. Guénard, *personal observation*). Similarly native species of the genera *Pheidole*, *Dorymyrmex* or *Forelius*, usually found in urban or open habitat [73], [75], and also considered as disturbance specialists [76], [77], have been totally absent from our sampling. Perhaps most telling, however, is our comparison of the sites with *P. chinensis* to a study of forests around the world by Ward [44], complemented by our own additional data (Table S3). Even when considered in the context of samples from forests around the world, the sites with *P. chinensis* were low in diversity, particularly given the total number of ants present therein. Finally, we note that anecdotally the sites invaded by *P. chinensis* appear to be “great anting grounds,” sites with thick litter, sticks and logs under a tall forest canopy where we would expect to find many native species. In the end, we cannot definitively reject the hypothesis that *P. chinensis* simply invades low diversity sites, but we find it very unlikely.

In light of our interpretation of the patterns we have observed, the presence and the potential spread of *P. chinensis* within natural habitat, particularly those being managed for conservation, may represent a threat to the local diversity and the functioning of ecosystems. The impact of *P. chinensis* on native ant community could also indirectly affect some of the ecological processes within forested habitats. In eastern North American forests, about a third of understory plants are ant dispersed [78], [79] and species from the *Aphaenogaster* genus, more specially *A. rudis*, have been clearly identified as the most important seed dispersers for myrmecochorous plants [78], [79], [80], [81], [82]. Abundance of *Aphaenogaster* species within forests of North Carolina appears to be correlated with the abundance of immature myrmecochores [83]. These plants are known to be very sensitive to disturbance [84] and to possess limited dispersal abilities and low germination rates [85]. Despite an anecdotal seed dispersal observation by *P. chinensis* in its native range [86]; it seems likely that the strong reduction or absence of the *Aphaenogaster* species may disrupt the population replacement of understory myrmecochorous plants. As another example of the potential consequences of the abundance of *P. chinensis*, the reduction of hypogaeic ants, the specialist predators on small arthropods, can be predicted to lead to a reduction of the top-down effects on the control of the small arthropods populations. Finally large population of *P. chinensis* may reduce termite abundance (J. Brightwell pers. obs.), with consequent effects on decomposition rates.

In conclusion, our study presents the first demonstration of invasion by an ant from the subfamily Ponerinae [21], [27]; moreover this invasion has occurred primarily within undisturbed habitats, most of them presently being managed for conservation. The large geographic distribution of *P. chinensis* over the east coast of the USA and its large scale consequences should be considered in more detail. This ant's influence may ramify widely because of its effects on human health and on native ant species and the processes they mediate, but also because of the extent to which this ant appears to break some of the “rules” of ant invasion. Many traits associated with invasion success in ants, such as monopolization of carbohydrate resources [6], supercoloniality and disturbance should be investigated in more details to understand at which extent *P. chinensis* fits the model developed for ant invasions. More than anything, the success of *P. chinensis*, may be evidence that when hundreds ant species are introduced each year from one region to another, many different ways exist to succeed.

## Supporting Information

Figure S1 Known distribution by county of *P. chinensis* in its introduced range on the East Coast of the USA.Counties where *P. chinensis* populations have been recorded appear in red on the map.
(8.95 MB TIF)

Table S1 Species richness, abundance and occurrence of the species collected with pitfall traps. Number of individuals and percentage of occurrence (in parenthesis) of each species as a function of how the study design was treated statistically and the presence or absence of *P. chinensis* in pitfall traps.
(0.10 MB DOC)

Table S2 Species richness, abundance and occurrence of the species collected with Winkler extractors. Number of individuals (and percentage of occurrences) of each species in Winkler bag extractions from sites with or without *P. chinensis*. Hypogaeic (subterranean) species are represented in bold.
(0.05 MB DOC)

Table S3 Species density and abundance of ants collected in areas with and without *P. chinensis*. Leaf litter ant species richness and abundance data were extracted from Ward [44], with the addition of data from the sites below. *P. chinensis* was absent for the last four sites presented in the table.
(0.04 MB DOC)

## References

[1] Pimentel D, Lach L, Zuniga R, Morrison D (2000). Environmental and economic costs of nonindigenous species in the United States.. BioScience.

[2] Pimentel D, McNair S, Janecka J, Wightman J, Simmonds C (2001). Economic and environmental threats of alien plant, animal, and microbe invasions.. Agricult Ecosys Environ.

[3] Pimentel D, Zuniga R, Morrison D (2005). Update on the environmental and economic costs associated with alien-invasive species in the United States.. Ecol Econ.

[4] Moller H (1996). Lessons for invasion theory from social insects.. Biol Conser.

[5] Lowe S, Browne M, Boudlejas S (2000). 100 of the world's worst invasive alien species.. Alien.

[6] Holway DA, Lach L, Suarez AV, Tsutsui ND, Case TJ (2002). The causes and consequences of ant invasions.. Annu Rev Ecol Syst.

[7] Allen CR, Epperson DM, Garmestani AS (2004). Red Imported Fire Ant impacts on wildlife: a decade of research.. Am Midl Nat.

[8] Ness JH, Bronstein JL (2004). The effects of invasive ants on prospective ant mutualists.. Biol Invasions.

[9] Lach L, Thomas ML (2008). Invasive ants in Australia: documented and potential ecological consequences.. Aust J Entomol.

[10] Le Breton J, Chazeau J, Jourdan H (2003). Immediate impacts of invasion by *Wasmannia auropunctata* (Hymenoptera: Formicidae) on native litter ant fauna in a New Caledonian rainforest.. Austral Ecol.

[11] Sarty M, Abbott K, Lester P (2007). Community level impacts of an ant invader and food mediated coexistence.. Insect Soc.

[12] Hoffmann BD, Andersen AN, Hill GJE (1999). Impact of an introduced ant on native rain forest invertebrates: *Pheidole megacephala* in monsoonal Australia.. Oecologia.

[13] Hoffmann B, Parr C (2008). An invasion revisited: the African big-headed ant (*Pheidole megacephala*) in northern Australia.. Biol Invasions.

[14] Human KG, Gordon DM (1997). Effects of Argentine ants on invertebrate biodiversity in northern California.. Conser Biol.

[15] Holway DA (1998). Effect of Argentine ant invasions on ground-dwelling arthropods in northern California riparian woodlands.. Oecologia.

[16] Holway DA (2005). Edge effects of an invasive species accross a natural ecological boundary.. Biol Conser.

[17] Suarez AV, Bolger DT, Case TJ (1998). Effects of fragmentation and invasion on native ant communities in coastal southern California.. Ecology.

[18] Sanders NJ, Gotelli NJ, Heller NE, Gordon DM (2003). Community disassembly by an invasive species.. Proc Am Acad Arts Sci.

[19] Lockwood JL, Purvis A. JLG, Brooks TM (2005). Predicting which species will become invasive: what's taxonomy got to do with it.. Phylogeny and conservation.

[20] Suarez AV, McGlynn TP, Tsutsui ND, Lach L. CP, Abbott K (2010). Biogeographic and taxonomic patterns of introduced ants.. Ant Ecology.

[21] Hölldobler B, Wilson EO (1990). The ants.

[22] McGlynn TP (1999). The worldwide transfer of ants: geographical distribution and ecological invasions.. J Biogeogr.

[23] Lester PJ (2005). Determinants for the successful establishment of exotic ants in New Zealand.. Divers Distrib.

[24] Suarez AV, Holway DA, Ward PS (2005). The role of opportunity in the unintentional introduction of nonnative ants.. Proc Natl Acad Sci USA.

[25] Boer P, Vierbergen B (2008). Exotic ants in the Netherlands (Hymenoptera: Formicidae).. Entomol Ber.

[26] Nelder MP, Paysen ES, Zungoli PA, Benson E (2006). Emergence of the introduced ant *Pachycondyla chinensis* (Formicidae: Ponerinae) as a public health threat in the Southeastern United States.. J Med Entomol.

[27] Peeters C, Choe JC, Crespi BJ (1997). Morphologically ‘primitive’ ants: comparative review of social characters, and the importance of queen-worker dimorphism.. The evolution of social behavior in insects and arachnids.

[28] Mac MJ, Opler PA, Puckett Haecker CE, Doran PD, Department of Interior USGS, editor (1998). Statues and trends of the nation's biological resources;.

[29] Smith MR (1934). Ponerine ants of the genus *Euponera* in the United States.. Ann Entomol Soc Am.

[30] Yashiro T, Matsuura K, Guenard B, Terayama M, Dunn RR (In prep.).

[31] Lee EK, Jeong KY, Lyu DP, Lee YW, Sohn JH (2009). Characterization of the major allergens of *Pachycondyla chinensis* in ant sting anaphylaxis patients.. Clin Exp Allergy.

[32] Kim SS, Park HS, Kim HY, Lee SK, Nahm DH (2001). Anaphylaxis caused by the new ant, *Pachycondyla chinensis*: demonstration of specific IgE and IgE-binding components.. J Allergy Clin Immunol.

[33] Bae GR, Lim HS, Kim BJ (1999). Epidemiologic survey on outbreak of dermatosis associated with ants, *Pachycondyla chinensis*.. Korean J Prev Med.

[34] Yun YY, Ko SH, Park JW, Hong CS (1999). Anaphylaxis to venom of the *Pachycondyla* species ant.. J Allergy Clin Immunol.

[35] Cho YS, Lee YM, Lee CK, Yoo B, Park HS (2002). Prevalence of *Pachycondyla chinensis* allergy in ant-infested area in Korea.. J Allergy Clin Immunol.

[36] Leath TM, Grier TJ, Jacobson RS, Fontana-Penn ME (2006). Anaphylaxis to *Pachycondyla chinensis*.. J Allergy Clin Immunol.

[37] Smith MR (1947). A generic and subgeneric synopsis of the United States ants, based on the workers.. Am Midl Nat.

[38] Peck SL, McQuaid B, Campbell CL (1998). Using ant species (Hymenoptera: Formicidae) as a biological indicator of agroecosystem condition.. Environ Entomol.

[39] MacGown JA (2009). The Asian needle ant, *Pachycondyla chinensis* Emery (Hymenoptera: Formicidae), reported from Alabama.. Midsouth Entomologist.

[40] Colwell RK (2009). EstimateS: Statistical estimation of species richness and shared species from samples.. http://purl.oclc.org/estimates.

[41] Ivanov K, Keiper J (2009). Effectiveness and biases of Winkler litter extractor and pitfall trapping for collecting ground-dwelling ants in northern temperate forests.. Environ Entomol.

[42] Bestelmeyer BT, Agosti D, Alonso LE, Brandão CRF, Brown WL, Agosti D, Majer JD, Alonso LE, Schultz TR (2000). Field techniques for the study of ground-dwelling ants: an overview, description, and evaluation.. Ants: standard methods for measuring and monitoring biodiversity.

[43] Ward PS (1987). Distribution of the introduced Argentine ant (*Iridomyrmex humilis*) in natural habitats of the Lower Sacramento Valley and its effects on the indigenous ant fauna.. Hilgardia.

[44] Ward PS, Agosti D, Majer JD, Alonso LE, Schultz TR (2000). Broad-scale patterns of diversity in leaf litter ant communities.. Ants: standard methods for measuring and monitoring biodiversity.

[45] Fowler HG (1992). Native ant simplification by introduction of an exotic ant following hydroelectric dam construction in Northeastern Brazil.. Cienc Cult.

[46] Porter SD, Savignano DA (1990). Invasion of polygyne fire ants decimates native ants and disrupts arthropod community.. Ecology.

[47] Heterick B (1997). The interaction between the coastal brown ant, *Pheidole megacephala* (Fabricius), and other invertebrate fauna of Mt Coot-tha (Bribane, Australia).. Aust J Ecol.

[48] Kennedy TA (1998). Patterns of an invasion by Argentine ants (*Linepithema humile*) in a riparian corridor and its effects on ant diversity.. Am Midl Nat.

[49] Oliveras J, Bas JM, Casellas D, Gomez C (2005). Numerical dominance of the Argentine ant vs native ants and consequences on soil resource searching in Mediterranean cork-oak forests (Hymenoptera: Formicidae).. Sociobiology.

[50] Sanders NJ (2004). Immediate effects of fire on the invasive Argentine ant, *Linepithema humile*.. Southwest Nat.

[51] Sanders NJ, Barton KE, Gordon DM (2001). Long-term dynamics of the distribution of the invasive Argentine ant, *Linepithema humile*, and native ant taxa in northern California.. Oecologia.

[52] Tillberg CV, Holway DA, LeBrun EG, Suarez AV (2007). Trophic ecology of invasive Argentine ants in their native and introduced ranges.. Proc Natl Acad Sci USA.

[53] Holway D (1998). Factors governing rate of invasion: a natural experiment using Argentine ants.. Oecologia.

[54] Helms KR, Vinson SB (2002). Widespread association of the invasive ant *Solenopsis invicta* with an invasive mealybug.. Ecology.

[55] Lach L (2003). Invasive ants: unwanted partners in ant-plant interactions?. Ann Mo Bot Gard.

[56] Davidson DW (1998). Resource discovery versus resource domination in ants: a functional mechanism for breaking the trade-off.. Ecol Entomol.

[57] Oliver TH, Pettitt H, Leather SR, Cook JM (2008). Numerical abundance of invasive ants and monopolisation of exudate-producing resources – a chicken and egg situation.. Insect Conserv Diver.

[58] Grover CD, Kay AD, Monson JA, Marsh TC, Holway DA (2007). Linking nutrition and behavioural dominance: carbohydrate scarcity limits aggression and activity in Argentine ants.. Proc R Entomol Soc London B Bio.

[59] Rowles AD, Silverman J (2009). Carbohydrate supply limits invasion of natural communities by Argentine ants.. Oecologia.

[60] Orivel J, Malherbe MC, Dejean A (2001). Relationship between pretarsus morphology and arboreal life in ponerine ants of the genus *Pachycondyla* (Formicidae: Ponerinae).. Ann Entomol Soc Am.

[61] Teranishi Y (1929). Habits and distributions of Japanese ants II.. Zool Mag.

[62] Matsuura K (2002). Colony-level stabilization of soldier head width for head-plug defence in the termite *Reticulermes speratus* (Isoptera: Rhinotermitidae).. Behav Ecol Sociobiol.

[63] Deheer CJ, Vargo EL (2004). Colony genetic organization and colony fusion in the termite *Reticulitermes flavipes* as revealed by foraging patterns over time and space.. Mol Ecol.

[64] Howard RW, Jones SC, Mauldin JK, Beal RH (1982). Abundance, distribution, and colony size estimates for *Reticulitermes* spp. (Isoptera: Rhinotermitidae) in Southern Mississippi. Abundance, distribution, and colony size estimates for *Reticulitermes* spp. (Isoptera: Rhinotermitidae) in Southern Mississippi.. Environ Entomol.

[65] Thorne BL, Traniello JFA, Adams ES, Bulmer M (1999). Reproductive dynamics and colony structure of subterranean termites of the genus *Reticulitermes* (Isoptera: Rhinotermitidae): a review of the evidence from behavioral, ecological, and genetic studies.. Ethol Ecol Evol.

[66] Baroni-Urbani CK, P.B. (1974). Patterns in the red imported fire ant settlement of a Louisiana pasture: some demographic parameters, interspecific competition and food sharing.. Environ Entomol.

[67] Helms KR, Vinson SB (2001). Coexistence of native ants with the red imported fire ant, *Solenopsis invicta*.. Southwest Nat.

[68] Morrison LW (2002). Long-term impacts of an arthropod-community invasion by the imported fire ant, *Solenopsis invicta*.. Ecology.

[69] Morrison LW, Porter SD (2003). Positive association between densities of the red imported fire ant, *Solenopsis invicta*, and generalized ant and arthropod diversity.. Environ Entomol.

[70] King JR, Tschinkel W (2006). Experimental evidence that the introduced fire ant, *Solenopsis invicta*, does not competitively suppress co-occurring ants in a disturbed habitat.. J Anim Ecol.

[71] King JR, Tschinkel WR (2008). Experimental evidence that human impacts drive fire ant invasions and ecological change.. Proc Natl Acad Sci USA.

[72] Stuble KL, Kirkman K, Carroll CR (2009). Patterns of abundance of fire ants and native ants in a native ecosystem.. Ecol Entomol.

[73] Carter WG (1962). Ant distribution in North Carolina.. J Elisha Mitchell Sci Soc.

[74] Nuhn TP, Wright CG (1979). An ecological survey of ants (Hymenoptera: Formicidae) in a landscaped suburban habitat.. Am Midl Nat.

[75] Carter WG (1962). Ants of the North Carolina Piedmont.. J Elisha Mitchell Sci Soc.

[76] Graham JH, Hughie HH, Jones S, Wrinn K, Krzysik AJ (2004). Habitat disturbance and the diversity and abundance of ants (Formicidae) in the Southeastern Fall-Line Sandhills.. J Insect Sci.

[77] Graham JH, Krzysik AJ, Kovacic DA, Duda JJ, Freeman DC (2008). Ant community composition across a gradient of disturbed military landscapes at Fort Benning, Georgia.. Southeast Nat.

[78] Handel SN, Fisch SB, Schatz GE (1981). Ants disperse a majority of herbs in a mesic forest community in NY State.. B Torrey Bot Club.

[79] Beattie AJ, Culver DC (1981). The guild of myrmecochores ants in the herbaceous flora of West Virginia forests.. Ecology.

[80] Culver DC, Beattie AJ (1978). Myrmecochory in *Viola*: dynamics of seed-ant interactions in some West Virginia species.. J Ecol.

[81] Zelikova TJ, Dunn RR, Sanders NJ (2008). Variation in seed dispersal along an elevational gradient in Great Smoky Mountains National Park.. Acta Oecol.

[82] Ness JH, Morin DF, Giladi I (2009). Uncommon specialization in a mutualism between a temperate herbaceous plant guild and an ant: are *Aphaenogaster* ants keystone mutualist?. Oikos.

[83] Mitchell CE, Turner MG, Pearson SM (2002). Effects of historical land use and forest patch size on myrmecochores and ant communities.. Ecol Appl.

[84] Duffy DC, Meier AJ (1992). Do Appalachian herbaceous understories ever recover from cleacutting?. Conser Biol.

[85] Struik GJ (1965). Growth patterns of some native annual and perennial herbs in southern Wisconsin.. Ecology.

[86] Ohnishi Y, Suzuki N, Katayama N, Teranishi S (2008). Seasonally different modes of seed dispersal in the prostrate annual, *Chamaesyce maculata* (L.) Small (Euphorbiaceae), with multiple overlapping generations.. Ecol Res.

