# Supplementary figures and images for: A New (Old), Invasive Ant in the Hardwood Forests of Eastern North America and Its Potentially Widespread Impacts
Source: PLoS One. 2010 Jul 21;5(7):e11614. doi: 10.1371/journal.pone.0011614 (PMC2908120; doi:10.1371/journal.pone.0011614)

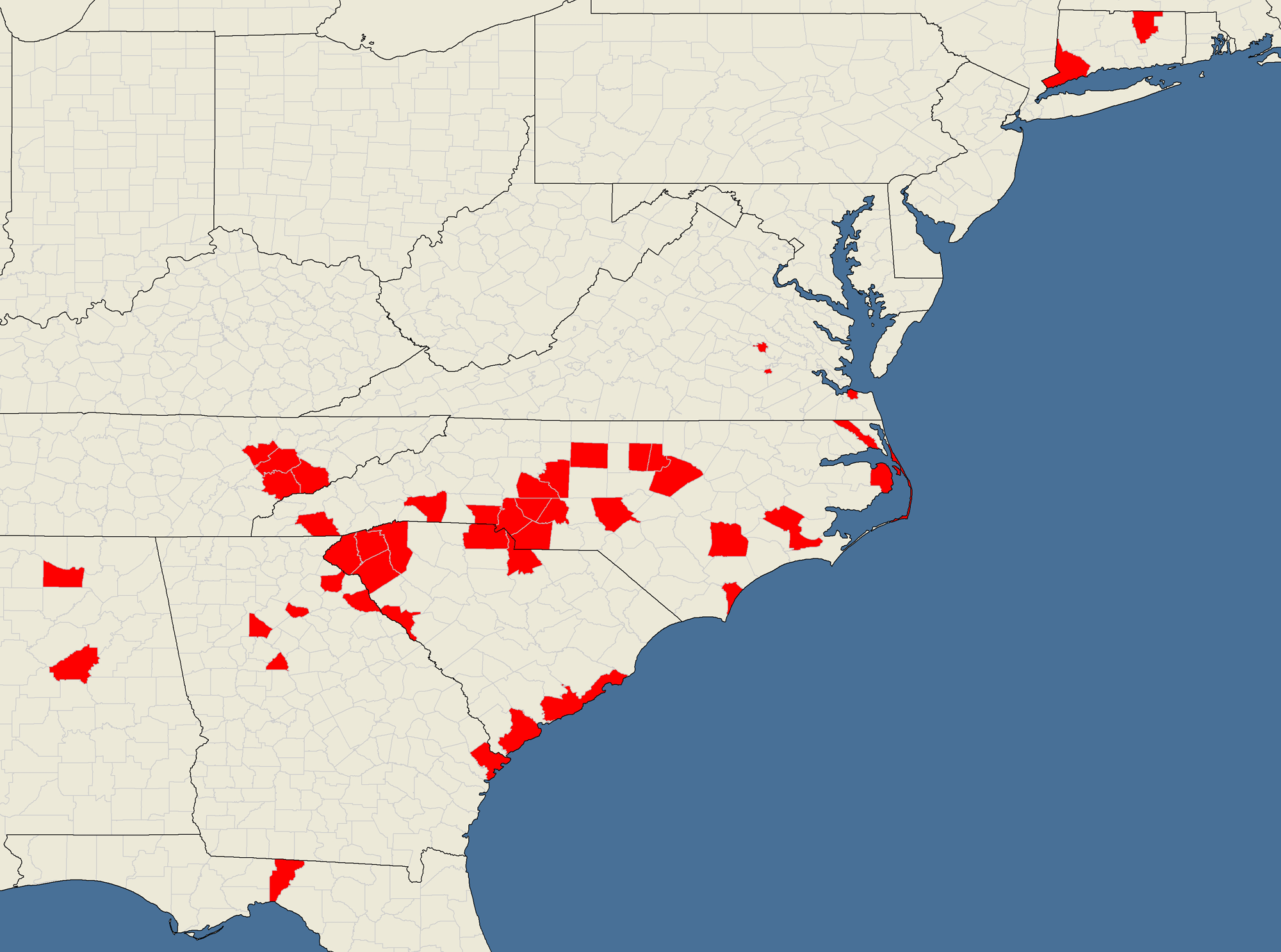

Supplement: Figure S1 — Known distribution by county of P. chinensis in its introduced range on the East Coast of the USA.Counties where P. chinensis populations have been recorded appear in red on the map. (8.95 MB TIF) [file pone.0011614.s001.tif]
