# Supplementary material for: A New (Old), Invasive Ant in the Hardwood Forests of Eastern North America and Its Potentially Widespread Impacts
Source: PLoS One. 2010 Jul 21;5(7):e11614. doi: 10.1371/journal.pone.0011614 (PMC2908120; doi:10.1371/journal.pone.0011614)
Supplement: Table S1 — Species richness, abundance and occurrence of the species collected with pitfall traps. Number of individuals and percentage of occurrence (in parenthesis) of each species as a function of how the study design was treated statistically and the presence or absence of P. chinensis in pitfall traps. (0.10 MB DOC) [file pone.0011614.s002.doc]

|  | **Match paired design** | | **Presence of *P. chinensis* design** | | **Total** |
| --- | --- | --- | --- | --- | --- |
|  | *P. chinensis* site (N=315) | No *P. chinensis* site (N=299) | With *P. chinensis* (N=306) | Without *P. chinensis* (N=308) |  |
| ***Pachycondyla chinensis*** | 7918 *(87.3)* | 117 *(10.4)* | 8035 *(100)* | 0 *(0)* | 8035 *(49.8)* |
| *Formica subserica* | 906 *(59)* | 889 *(40.5)* | 883 *(58.2)* | 912 *(41.9)* | 1795 *(50)* |
| *Aphaenogaster carolinensis* | 271 *(19.4)* | 1435 *(84.6)* | 149 *(14.7)* | 1557 *(87.3)* | 1706 *(51.1)* |
| *Solenopsis carolinensis* | 121 *(4.4)* | 503 *(30.4)* | 119 *(5.2)* | 505 *(28.9)* | 624 *(17.1)* |
| *Lasius alienus* | 64 *(3.5)* | 174 *(10.7)* | 50 *(3.3)* | 188 *(10.7)* | 238 *(7)* |
| *Camponotus castaneus* | 105 *(21)* | 87 *(20.7)* | 96 *(19.6)* | 96 *(22.1)* | 192 *(20.8)* |
| *Camponotus chromadoides* | 162 *(25.7)* | 26 *(5)* | 157 *(25.5)* | 31 *(5.8)* | 188 *(15.6)* |
| *Aphaenogaster fulva* | 28 *(2.5)* | 150 *(15.7)* | 13 *(1.6)* | 165 *(16.2)* | 178 *(9)* |
| *Camponotus pennsylvanicus* | 142 *(15.9)* | 36 *(8.4)* | 149 *(18)* | 29 *(6.5)* | 178 *(12.2)* |
| *Lasius umbratus* | 9 *(0.6)* | 137 *(1.3)* | 9 *(0.7)* | 137 *(1.3)* | 146 *(1)* |
| *Formica pallidefulva* | 75 *(15.2)* | 46 *(11)* | 59 *(13.7)* | 62 *(12.7)* | 121 *(13.2)* |
| *Camponotus americanus* | 77 *(11.4)* | 40 *(6)* | 87 *(11.8)* | 30 *(5.8)* | 117 *(8.8)* |
| *Crematogaster vermiculata* | 71 *(9.5)* | 38 *(8.4)* | 53 *(6.9)* | 56 *(11)* | 109 *(9)* |
| *Myrmecina americana* | 17 *(3.8)* | 90 *(15.4)* | 11 *(2.6)* | 96 *(16.2)* | 107 *(9.4)* |
| *Lasius flavus* | 31 *(5.4)* | 64 *(10.7)* | 34 *(5.9)* | 61 *(10.1)* | 95 *(8)* |
| *Nylanderia faisonensis* | 10 *(1.6)* | 63 *(8.7)* | 7 *(1.3)* | 66 *(8.8)* | 73 *(5)* |
| *Aphaenogaster rudis* | 0 *(0)* | 51 *(2.3)* | 0 *(0)* | 51 *(2.3)* | 51 *(1.1)* |
| *Temnothorax curvispinosus* | 11 *(2.2)* | 37 *(8.7)* | 6 *(1.6)* | 42 *(9.2)* | 48 *(5.4)* |
| *Aphaenogaster miamiana* | 3 *(0.3)* | 43 *(5)* | 6 *(0.7)* | 40 *(4.5)* | 46 *(2.6)* |
| *Ponera pennsylvanica* | 14 *(3.5)* | 30 *(9)* | 10 *(2.6)* | 34 *(9.7)* | 44 *(6.2)* |
| *Aphaenogaster lamellidens* | 6 *(1.6)* | 30 *(3.3)* | 5 *(1.3)* | 31 *(3.6)* | 36 *(2.4)* |
| *Monomorium minimum* | 23 *(3.8)* | 13 *(2.7)* | 9 *(2.3)* | 27 *(4.2)* | 36 *(3.3)* |
| *Lasius (Acanthomyops) sp1* | 0 *(0)* | 32 *(0.3)* | 0 *(0)* | 32 *(0.3)* | 32 *(0.2)* |
| *Trachymyrmex septentrionalis* | 24 *(2.2)* | 2 *(0.3)* | 20 *(1.6)* | 6 *(1)* | 26 *(1.3)* |
| *Nylanderia* sp*.(parvula* cplx*)* | 3 *(0.6)* | 20 *(4.7)* | 3 *(0.7)* | 20 *(4.5)* | 23 *(2.6)* |
| *Aphaenogaster treatae* | 2 *(0.6)* | 19 *(2.3)* | 4 *(1)* | 17 *(1.9)* | 21 *(1.5)* |
| *Aphaenogaster tennesseensis* | 0 *(0)* | 17 *(2)* | 0 *(0)* | 17 *(1.9)* | 17 *(1)* |
| *Crematogaster lineolata* | 0 *(0)* | 17 *(1.7)* | 0 *(0)* | 17 *(1.6)* | 17 *(0.8)* |
| *Lasius interjectus* | 16 *(0.6)* | 0 *(0)* | 7 *(0.3)* | 9 *(0.3)* | 16 *(0.3)* |
| *Temnothorax tuscaloosae* | 0 *(0)* | 16 *(3.3)* | 3 *(1)* | 13 *(2.3)* | 16 *(1.6)* |
| *Formica gr. sanguinea* | 12 *(2.2)* | 3 *(1)* | 7 *(2)* | 8 *(1.3)* | 15 *(1.6)* |
| *Prenolepis imparis* | 12 *(1.6)* | 3 *(1)* | 4 *(1)* | 11 *(1.6)* | 15 *(1.3)* |
| *Crematogaster ashmeadi* | 9 *(2.2)* | 4 *(1.3)* | 9 *(2.3)* | 4 *(1.3)* | 13 *(1.6)* |
| *Camponotus nearticus* | 9 *(1.9)* | 2 *(0.7)* | 8 *(1.6)* | 3 *(1)* | 11 *(1.3)* |
| *Neivamyrmex nigrescens* | 5 *(0.3)* | 3 *(1)* | 0 *(0)* | 8 *(1.3)* | 8 *(0.7)* |
| *Camponotus subarbatus* | 6 *(1.9)* | 0 *(0)* | 6 *(2)* | 0 *(0)* | 6 *(1)* |
| *Lasius sublager* | 0 *(0)* | 5 *(0.3)* | 0 *(0)* | 5 *(0.3)* | 5 *(0.2)* |
| *Tapinoma sessile* | 5 *(1.6)* | 0 *(0)* | 4 *(1.3)* | 1 *(0.3)* | 5 *(0.8)* |
| *Pyramica rostrata* | 0 *(0)* | 4 *(1.3)* | 0 *(0)* | 4 *(1.3)* | 4 *(0.7)* |
| *Brachmyrmex depilis* | 3 *(0.3)* | 0 *(0)* | 3 *(0.3)* | 0 *(0)* | 3 *(0.2)* |
| *Pyramica ornata* | 0 *(0)* | 3 *(1)* | 0 *(0)* | 3 *(1)* | 3 *(0.5)* |
| *Temnothorax texanus* | 0 *(0)* | 3 *(1)* | 0 *(0)* | 3 *(1)* | 3 *(0.5)* |
| *Amblyopone pallipes* | 0 *(0)* | 2 *(0.7)* | 0 *(0)* | 2 *(0.6)* | 2 *(0.3)* |
| *Hypoponera opacior* | 1 *(0.3)* | 1 *(0.3)* | 1 *(0.3)* | 1 *(0.3)* | 2 *(0.3)* |
| *Pyramica ohioensis* | 0 *(0)* | 2 *(0.7)* | 0 *(0)* | 2 *(0.6)* | 2 *(0.3)* |
| *Strumigenys louisianae* | 1 *(0.3)* | 1 *(0.3)* | 1 *(0.3)* | 1 *(0.3)* | 2 *(0.3)* |
| *Temnothorax longispinosus* | 1 *(0.3)* | 1 *(0.3)* | 0 *(0)* | 2 *(0.6)* | 2 *(0.3)* |
| *Camponotus obliquus* | 1 *(0.3)* | 0 *(0)* | 1 (0.3) | 0 (0) | 1 *(0.2)* |
| *Crematogaster pilosa* | 0 *(0)* | 1 *(0.3)* | 0 *(0)* | 1 *(0.3)* | 1 *(0.2)* |
| *Pyramica pilinasis* | 0 *(0)* | 1 *(0.3)* | 0 *(0)* | 1 *(0.3)* | 1 *(0.2)* |
| *Temnothorax pergandei* | 0 *(0)* | 1 *(0.3)* | 0 *(0)* | 1 *(0.3)* | 1 *(0.2)* |
| *Temnothorax cf. shaumii* | 0 *(0)* | 1 *(0.3)* | 0 *(0)* | 1 *(0.3)* | 1 *(0.2)* |
| ***Total species*** | **37** | **48** | **36** | **49** | **52** |
| ***Total abundance*** | **10174** | **4263** | **10028** | **4409** | **14437** |
| ***Total native species abundance*** | **2256** | **4146** | **1993** | **4409** | **6402** |
