# Supplementary material for: A New (Old), Invasive Ant in the Hardwood Forests of Eastern North America and Its Potentially Widespread Impacts
Source: PLoS One. 2010 Jul 21;5(7):e11614. doi: 10.1371/journal.pone.0011614 (PMC2908120; doi:10.1371/journal.pone.0011614)
Supplement: Table S2 — Species richness, abundance and occurrence of the species collected with Winkler extractors. Number of individuals (and percentage of occurrences) of each species in Winkler bag extractions from sites with or without P. chinensis. Hypogaeic (subterranean) species are represented in bold. (0.05 MB DOC) [file pone.0011614.s003.doc]

|  | *P. chinensis* site  (N=4) | No *P. chinensis* site (N=4) | **Total** (N=8) |
| --- | --- | --- | --- |
| *Pachycondyla chinensis* | 560 *(100)* | 0 (0) | 560 *(50)* |
| *Nylanderia* sp*. (parvula* cplx) | *0 (0)* | *284 (50)* | 284 *(25)* |
| ***Solenopsis carolinensis*** | *0 (0)* | *179 (50)* | 179 (50) |
| *Aphaenogaster carolinensis* | *0 (0)* | *154 100)* | 154 (50) |
| *Temnothorax curvispinosus* | *0 (0)* | *130 (100)* | 130 (50) |
| *Temnothorax tuscaloosae* | *0 (0)* | *109 (50)* | 109 (25) |
| ***Pyramica rostrata*** | *0 (0)* | *106 (50)* | 106 (25) |
| ***Ponera pennsylvanica*** | *0 (0)* | *91 (100)* | 91 (50) |
| ***Myrmecina americana*** | *0 (0)* | *83 (100)* | 83 (50) |
| *Lasius alienus* | *0 (0)* | *62 (50)* | 62 (25) |
| ***Pyramica ohioensis*** | *0 (0)* | *56 (25)* | 56 (12.5) |
| *Formica subsericea* | *15 (50)* | *27 (50)* | 42 (50) |
| ***Pyramica ornata*** | *0 (0)* | *27 (75)* | 27 (37.5) |
| *Aphaenogaster fulva* | *0 (0)* | *7 (25)* | 7 (25) |
| ***Pyramica angulata*** | *0 (0)* | *6 (25)* | 6 (12.5) |
| ***Pyramica pergandei*** | *0 (0)* | *6 (25)* | 6 (12.5) |
| ***Amblyopone pallipes*** | *0 (0)* | *3 (50)* | 3 (25) |
| *Aphaenogaster rudis* | *0 (0)* | *3 (25)* | 3 (12.5) |
| ***Lasius flavus*** | *0 (0)* | *3 (25)* | 3 (12.5) |
| *Crematogaster vermiculata* | *0 (0)* | *2 (25)* | 2 (12.5) |
| ***Proceratium silaceum*** | *0 (0)* | *2 (25)* | 2 (12.5) |
| *Formica pallidefulva* | *0 (0)* | *2 (50)* | 2 (25) |
| ***Strumigenys louisianae*** | *0 (0)* | *2 (25)* | 2 (12.5) |
| *Camponotus castaneus* | 1 (25) | 0 (0) | 1 (12.5) |
| *Camponotus pennsylvanicus* | 0 (0) | 1 (25) | 1 (12.5) |
| *Camponotus subbarbatus* | 0 (0) | 1 (25) | 1 (12.5) |
| *Tapinoma sessile* | 0 (0) | 1 (1) | 1 (12.5) |
| ***Total species*** | **3** | **25** | **27** |
| ***Total abundance*** | **576** | **1347** | **1923** |
| ***Total native species abundance*** | **16** | **1347** | **1363** |
