# Supplementary material for: A New (Old), Invasive Ant in the Hardwood Forests of Eastern North America and Its Potentially Widespread Impacts
Source: PLoS One. 2010 Jul 21;5(7):e11614. doi: 10.1371/journal.pone.0011614 (PMC2908120; doi:10.1371/journal.pone.0011614)
Supplement: Table S3 — Species density and abundance of ants collected in areas with and without P. chinensis. Leaf litter ant species richness and abundance data were extracted from Ward [44], with the addition of data from the sites below. P. chinensis was absent for the last four sites presented in the table. (0.04 MB DOC) [file pone.0011614.s004.doc]

| **Country** | **Latitude** | **Longitude** | **Elevation (m)** | **Date** | **Species density** | **Abundance** |
| --- | --- | --- | --- | --- | --- | --- |
| Japan | 34.44 | 133.81 | 120 | 19/06/09 | 8 | 64 |
| Japan | 34.56 | 133.97 | 230 | 19/06/09 | 17 | 419 |
| Japan | 34.72 | 133.62 | 320 | 20/06/09 | 15 | 1031 |
| Japan | 34.72 | 133.60 | 370 | 20/06/09 | 14 | 652 |
| Japan | 34.69 | 133.92 | 50 | 21/06/09 | 17 | 932 |
| USA | 35.72 | -78.68 | 90 | 03/07/09 | 2 | 228 |
| USA | 35.72 | -78.68 | 90 | 10/07/09 | 3 | 71 |
| USA | 35.72 | -78.68 | 90 | 24/07/09 | 1 | 188 |
| USA | 35.72 | -78.68 | 90 | 24/07/09 | 1 | 89 |
| USA | 35.72 | -78.68 | 90 | 03/07/09 | 8 | 186 |
| USA | 35.72 | -78.68 | 90 | 10/07/09 | 15 | 389 |
| USA | 35.72 | -78.68 | 90 | 24/07/09 | 13 | 547 |
| USA | 35.72 | -78.68 | 90 | 24/07/09 | 11 | 225 |
